# Editorial commentary on the special section on cancer research

**DOI:** 10.7555/JBR.40.20268002

**Published:** 2026-05

**Authors:** Editorial Board

The four original articles in this special section explore diverse aspects of cancer biology, ranging from molecular mechanisms and tumor heterogeneity to behavioral risk prediction, thereby highlighting the power of integrating experimental biology with computational approaches.

Wang *et al*^[1]^ reveal that GPC6 promotes SHH-subgroup medulloblastoma by enhancing Hedgehog signaling through dual mechanisms. GPC6 acts as a co-receptor that supports signal transduction and promotes ciliogenesis—a critical step for SHH reception—while also facilitating SHH ligand secretion *via* extracellular vesicles. Functional assays confirmed that GPC6 drives the proliferation, migration, and invasion of medulloblastoma cells. These findings position GPC6 as a promising therapeutic target for this aggressive pediatric brain tumor.

Zhao *et al*^[2]^ integrated single-cell and bulk RNA sequencing to compare lung adenocarcinoma in smokers and never-smokers. Smoking-associated cancer cells exhibited greater aggressiveness and advanced pseudotime trajectories, correlating with poorer prognosis. Notably, immunosuppressive CXCL10^+^ macrophages were enriched in smokers and expressed multiple immune checkpoint molecules, with the LGALS9-HAVCR2 axis emerging as a potential immunotherapeutic target. In contrast, never-smokers displayed reduced anti-tumor cytotoxicity and an inflammatory microenvironment, suggesting distinct oncogenic mechanisms that may contribute to their suboptimal response to immunotherapy.

Xu *et al*^[3]^ uncovered a novel piRNA-mediated mechanism in colorectal cancer. piR-61298 was significantly upregulated in tumor tissues and directly bound to the deubiquitinase USP10 in the cytoplasm, impairing its ability to deubiquitinate and stabilize p53. This interaction resulted in proteasomal degradation of p53 and reduced p21 expression, thereby promoting proliferation, migration, and tumor growth in xenograft models. piR-61298 thus represents a potential therapeutic target and diagnostic biomarker.

Elzein *et al*^[4]^ applied logistic regression combined with principal component analysis and L1 regularization to predict cervical cancer risk based on behavioral factors. Using a UCI dataset consisting of 72 instances and 19 attributes, their model achieved 97.2% accuracy and an area under the curve (AUC) of 98.1%, substantially outperforming other classifiers including decision trees and XGBoost. SHAP analysis identifies contraceptive use and smoking as the strongest behavioral determinants. This simple and interpretable analytical pipeline offers a practical tool for low-resource settings where advanced screening methods remain unavailable.

Together, these studies advance our understanding of cancer progression and open new avenues for precision diagnosis and targeted therapy.
